# Effects of crossing distance on components of fitness in two rare rock plants

**DOI:** 10.1111/plb.70297

**Published:** 2026-10-07

**Authors:** G. Colling, L. Decanter, D. Matthies

**Affiliations:** ^1^ Plant Population Biology Musée national d'histoire naturelle Luxembourg Luxembourg; ^2^ Fondation Faune‐Flore Musée national d'histoire naturelle Luxembourg Luxembourg; ^3^ Department of Biology University of Marburg Marburg Germany

**Keywords:** Genetic load, heteroploid crossing, hybrid, outbreeding depression, polyploidy, reproductive isolation, Saxifraga

## Abstract

- To alleviate negative effects of genetic drift on rare plants, it has been suggested to artificially increase gene flow between isolated populations and create new populations by mixing different provenances to maximize genetic variation. However, there is also a risk of outbreeding depression.
- We studied the effects of cross type on components of fitness in two closely related taxa, the rare rock plants *Saxifraga rosacea* ssp. rosacea (octoploid) and *S. rosacea* ssp. *sponhemica* (hexaploid), a Central European endemic. Using plants from 30 populations, we carried out within‐population crosses (WPC), between‐population crosses (BPC) and between‐taxon crosses (BTC), and measured performance over two generations in a common garden. We also studied backcrosses (BTC × WPC).
- Seed yield of BPC and survival of offspring were higher than that of both WPC and BTC, indicating genetic load in the isolated rock populations of both taxa. However, BPC offspring showed outbreeding depression for reproduction, while BTC offspring was nearly completely sterile, and backcrosses hardly produced any seeds. The mass and germination of the seeds produced by the F_1_ and the F_2_, and survival of the F_2_ were not negatively affected by BPC and BTC.
- Our results suggest caution in increasing gene flow between long‐term isolated populations or mixing seeds for restoration projects. The nearly complete infertility of the F_1_‐plants resulting from BTC strongly supports the notion that management measures resulting in heteroploid crosses should be avoided. Moreover, the sterility of the hybrids suggests that the two taxa should be treated as separate species.

## Graphical abstract

Crossing experiments between populations of the rare rock species *Saxifraga rosacea* ssp. *rosacea* (8×) and *S. rosacea* ssp. *sponhemica* (6×) resulted in heterosis for some traits and outbreeding depression for others. Crossing the two subspecies resulted in sterile hybrids indicating that mixing populations of rare species is not always beneficial.

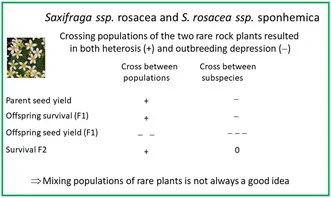

## INTRODUCTION

Due to anthropogenic destruction and fragmentation of habitats, populations of many plant species are decreasing in size and are becoming more isolated (Aguilar *et al*. 2019). Smaller and more isolated populations have an increased risk of extinction due to environmental stochasticity (Matthies *et al*. 2004) but are also affected more strongly by genetic drift, which results in an increase in the frequency of homozygotes and a loss of genetic diversity (Fischer & Matthies 1998; Colling *et al*. 2010; González *et al*. 2020; Bohm *et al*. 2025). Such genetic erosion may cause a loss of overall fitness and thus an increased extinction risk in the short term, but also a reduced ability to adapt to environmental change (Kéry *et al*. 2000; Reed 2005), which increases the long‐term risk of extinction.

To rescue populations from these threats, it has been proposed to artificially increase the gene flow between populations by the translocation of seeds or young plants as part of conservation management (Aitken & Whitlock 2013; Waller 2015; Whiteley *et al*. 2015). Introducing new genotypes into fragmented populations affected by genetic erosion can result in increased individual and population fitness (heterosis) by masking deleterious alleles (Oakley & Winn 2012; Van Rossum *et al*. 2020). Moreover, admixture can increase the genetic variation available for adaptation to local conditions and to changes in the abiotic or biotic environment (Verhoeven *et al*. 2011; Walisch *et al*. 2012; Sacristán‐Bajo *et al*. 2025). Similar arguments can be brought forward for using seed mixtures from several populations when creating new populations to reduce the risk of regional extinction (Sheridan & Karowe 2000; Brisset *et al*. 2025).

However, artificially increasing the gene flow between isolated populations can also result in reduced individual and population fitness. Local genotypes may have been selected by local conditions, and non‐local genotypes or hybrids could be maladapted to these conditions (Crémieux *et al*. 2010). Moreover, hybridization between populations could result in the breakup of coadapted gene complexes, in particular, in the F_2_ and later generations (Edmands 2007), resulting in a reduced fitness of offspring from between‐population crosses compared to that from within‐population crosses (outbreeding depression). However, few studies have investigated effects of crossing type beyond the first generation (Erickson & Fenster 2006; Etterson *et al*. 2007).

Understanding the relative importance of heterosis and outbreeding depression over multiple generations is a fundamental question that needs to be addressed empirically in conservation biology (Pickup *et al*. 2013). It has been suggested that the advantages of increased genetic diversity through outcrossing within species more than outweigh the possible negative effects of outbreeding depression if certain conditions are met: Populations should not have been isolated for more than 500 years, should inhabit similar environments or if they inhabit substantially different environments should only have been isolated for less than 20 generations (Frankham *et al*. 2011). Others have argued that admixture will be beneficial in most cases (Bossuyt 2007; Bürli *et al*. 2024), but there is no general consensus (Bell *et al*. 2019).

Particularly problematic can be the introduction of a different cytotype of a species into populations, which may result in cross ploidy hybridization and odd ploidy levels (Frankham *et al*. 2011). Seed set from cross ploidy pollinations is usually strongly reduced, the fitness of progeny low, and if hybrids are formed, they are sterile (Ramsey & Schemske 1998; Schmidt‐Lebuhn *et al*. 2018). Several authors have thus warned against the potentially negative consequences of interploidy crosses and demanded to test for intraspecific ploidy variation before mixing material from multiple populations in restoration projects (Severns & Liston 2008; Frankham *et al*. 2011; Kramer *et al*. 2018). However, a recent review found considerable variation in the effects of interploidy crosses among species (Salony *et al*. 2025), and recently several studies have indicated that cross ploidy crosses in natural populations of many species are frequent and that within population variation in ploidy levels is maintained (Kolář *et al*. 2017; Bartolić *et al*. 2024; Urfus *et al*. 2025). It has been suggested that high ploidy level crosses in particular may not have strong negative consequences for progeny fitness (Sonnleitner *et al*. 2013). Moreover, backcrosses between hybrids and their parent genotypes could provide an escape from hybrid sterility (Bartolić *et al*. 2024). However, until now, very few studies have investigated the effects of interploidy crosses between cytotypes with ploidy levels higher than tetraploid, or the success of backcrosses.

We experimentally studied the effects of crossing distance on two rare rock taxa, *S. rosacea* ssp. *rosacea* D.A. Webb and *S. rosacea* ssp. *sponhemica* (C.C. Gmel.) D.A. Webb. These taxa have been defined as subspecies of *S. rosacea* by Webb (1963) but had previously been considered to be two separate species, *S. rosacea* Moench and *S. sponhemica* C.C.Gmel. The two taxa have different ploidy levels, *S. rosacea* ssp. *rosacea* is octoploid and *S. rosacea* ssp. *sponhemica* is hexaploid (Decanter *et al*. 2020). *S. rosacea* ssp. *sponhemica* is a very rare central European endemic, while *S. rosacea* ssp. rosacea is rare in Central Europe, but also occurs in Scandinavia and Iceland (Webb 1963). Both taxa have been rare in the past because of the rarity of their habitats, but are also increasingly negatively impacted by the partial or total anthropogenic destruction of their populations (Decanter 2021).

The aim of this study was to investigate the effects of cross type on the performance of two generations of the two *Saxifraga* taxa with different ploidy levels. We performed crosses within populations, between populations and between the two different taxa, and carried out backcrosses between cross taxon hybrids and their parent taxa. We recorded seed production, seed mass, germination, survival and cumulative fitness over two generations. The different pollination treatments simulated which crosses would occur in new populations founded (i) by offspring from single populations, (ii) by admixture of offspring from multiple populations of a single taxon, or (iii) by mixing offspring from populations of the two taxa with different cytotypes. In addition, (iv) we studied the effects of backcrosses between interploidy hybrids and one of the parent taxa.

We address the following questions: (1) Would artificially increasing gene flow between populations result in heterosis or in outbreeding depression? (2) Do the effects of crossing type differ among life history traits and generations? (3) Are the two taxa reproductively isolated and should they be considered different species?

## MATERIAL AND METHODS

### Study species

*Saxifraga rosacea* MOENCH is a perennial plant species with a large distribution area in Europe. Webb & Gornall (1989) described three subspecies: *S. rosacea* ssp. *rosacea*, *S. rosacea* ssp. *sponhemica* and *S. rosacea* sbsp. *hartii*, which is only known from Arranmore Island in Ireland. In the present study, we focused on the first two subspecies, named for brevity *Saxifraga rosacea* and *Saxifraga sponhemica* in the following. Although the two *Saxifraga* taxa grow in similar habitats such as screes, stony slopes or walls, they differ in their ecological niche (Decanter *et al*. 2020) and no overlaps in their distribution areas are known. *S. rosacea* has a disjunct distribution and occurs in the French Jura, Central and Eastern Germany, the Faroe Islands, Western Ireland, Norway, Sweden, Finland and Iceland. *S. sponhemica* has a much smaller disjunct distribution and occurs in the Czech Republic, the Ardennes‐Eifel, the Hunsrück and the Jura regions (Decanter *et al*. 2020).

The two *Saxifraga* subspecies have a similar general morphology and reproductive system. The plants form cushions consisting of 1 to 600 rosettes. Floral stems grow from the rosette apex and develop small white protandrous flowers grouped into simple cymes. Plants flower from April to July in continental Europe. Flowers are self‐compatible and pollinated by a wide range of insect species such as Diptera (Muscidae and Syrphidae), Apidae and Coleoptera (Webb & Gornall 1989; Walisch *et al*. 2015) and produce small capsules (c. 3.8 × 4.7 mm) consisting of two locules which may contain over 200 seeds (c. 0.8 × 0.5 mm, mass c. 30 μg).

The two subspecies are taxonomically distinguished by their leaf morphology. The lobes of the leaves of *S. rosacea* are obtuse, acute or shortly mucronate with trichomes that are predominantly glandular, whereas the segments of the leaves of *S. sponhemica* are apiculate, narrow and have trichomes that are mostly non‐glandular (Webb & Gornall 1989). The two subspecies are also genetically differentiated (Webb & Gornall 1989; Decanter *et al*. 2020), as they have different cytotypes: *S. rosacea* plants are octoploid (8× = 64), while *S. sponhemica* plants are hexaploid (6× = 48).

### Sampling of parent plants

We collected seeds or rosettes in 15 populations of *S. rosacea* and 15 populations of *S. sponhemica* (Table S1) from 3 to 14 different plants per population depending on population size and available ripe seeds. Seeds were stored at 6°C in paper bags with silica gel until they were placed for germination (spring 2007) on moist filter paper in Petri dishes in an incubator (IPP260plus—Memmert GmbH + Co.KG, Büchenbach, Germany) at 20°C with a 16 h day and 8 h night regime. When the seedlings had reached a size of about 1 cm, they were transplanted to soaked peat pellets (38 mm) (Jiffy, Jiffy Group, Kristiansand, Norway) and kept in a growth chamber at c. 20°C under a 12 h day/12 h night light regime provided by fluorescent tubes (Sylvania®, Gro‐lux F58W/T8, Erlangen, Germany). Rosettes collected in the field were immediately placed on wet cotton in individual plastic sampling tubes and then transplanted to soaked peat pellets and kept under the same conditions as the seedlings. In September 2009, all plants were transferred to pots of 11 cm diameter filled with low‐nutrient soil (138 mg/L N, 108 mg/L P_2_O_2_, 158 mg/L K_2_O) and kept in the garden of the National Museum of Natural History of Luxembourg until the start of the crossing experiment. In the following, these plants are referred to as the parent generation (F_0_) plants (Fig. 1).

**Fig. 1:**
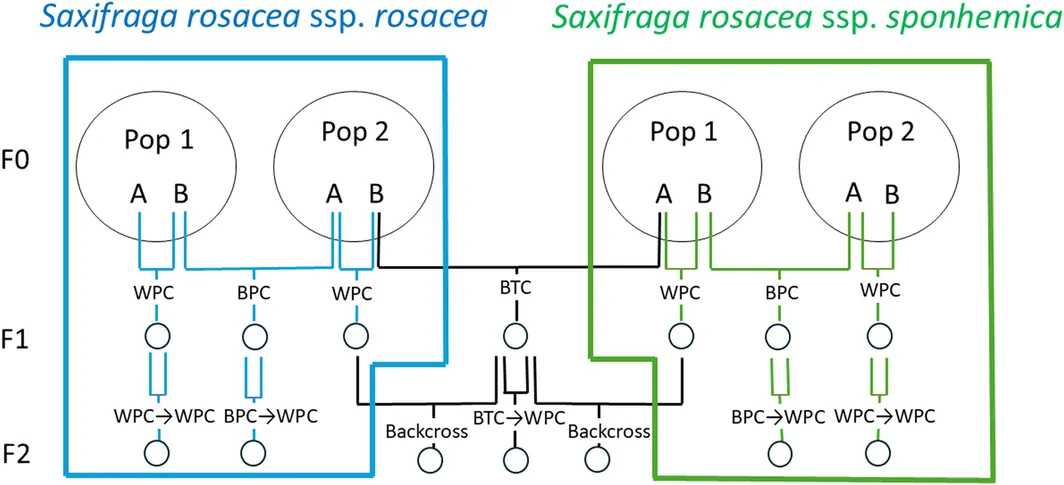
Schematic diagram showing the various crossing treatments over two generations of *S. rosacea* and *S. sponhemica*. WPC—within‐population cross; BPC—between‐population cross; BTC—between‐taxon cross; Backcross—cross between F_1_‐plants that had been produced by within‐population crosses, with plants that had been produced by between‐cytotype crosses, and one of whose parents was from the same population as the WPC‐individual.

### Crossing experiment

To create the F_1_‐generation, three types of crosses were applied in spring 2010 to the F_0_ individuals (Fig. 1): (1) Within‐Population Crosses (WPC) were carried out by pollinating a flower with pollen from another plant from the same population, (2) Between‐Population Crosses (BPC) by pollinating a flower with pollen from another plant from a different population of the same taxon and (3) Between‐Taxon Crosses (BTC) by pollinating a flower with pollen from a plant of the other taxon (Data S1). All 144 crossings were carried out in a glasshouse sealed with a mosquito net to keep natural pollinators out. The position of all plants was randomized every week.

To avoid self‐pollination, receiver flowers were emasculated before maturation of their anthers. Hand pollination was performed by gently rubbing two anthers from the donor flower across each ripe stigma of the receiver flower. Ripe fruits were collected and separately stored in small paper bags and dried at room temperature. Developed seeds were counted and stored at 6°C.

In autumn 2012, fruits from each crossing that had developed at least 20 ripe seeds were selected and 20–40 seeds from each fruit were weighed as a bulk sample and mean seed mass calculated. Because seeds of the two *Saxifraga* taxa are very small, the mean mass of seeds from fruits with less than 20 seeds could not be reliably determined. The selected seeds (n = 2740) were put for germination on moist filter paper in Petri dishes, placed in an incubator, and seedlings were raised under the same conditions as the F_0_ plants. The position of each Petri dish was randomized in the incubator every 3 days, and the number of germinated seeds was recorded. In spring 2013, seedlings (n = 1871) were individually transferred into pots (11 cm diameter) filled with low‐nutrient soil and placed in the experimental garden of the museum. In summer 2014, the survival of plants was recorded.

To obtain the second generation, 69 descendants from the different F_1_‐crosses were used as parent plants in spring 2014 to carry out four pollination treatments (Fig. 1): (1) The F_2_ WPC‐plants (WPC → WPC) were obtained by crossing individuals from the same F_1_‐population, (2) the F_2_ BPC‐plants (BPC → WPC) were obtained by crossing F_1_‐offspring from each BPC, (3) the F_2_ BTC‐plants (BTC → WPC) were obtained by crossing F_1_‐individuals from BTC and (4) backcross plants (WPC/BTC → Backcross) were obtained by crossing offspring from WPC‐crosses with offspring from BTC crosses, one of whose parents was from the same population as the WPC‐individual.

Pollination of the F_1_‐plants, storage and measurements of their seeds were performed as in the first part of the experiment. In autumn 2014, seeds (n = 2604) produced by the F_1_‐plants were germinated following the protocol for the previous generations. When the seedlings (n = 2005) had reached a diameter of c. 1 cm, they were transplanted individually into peat pellets and kept under the usual conditions. Survival of the plants was recorded in summer 2015.

### Statistical analyses

We analysed the effects of mother species, the pollination treatments (WPC, BPC, BTC) and their interaction on the following traits of the parent generation: the proportion of flowers of the parent plants developing into a fruit, the number of seeds per fruit and the mean mass of the individual seeds. We also analysed the effects of the factors of influence on the proportion of seeds that germinated and the survival of the F_1_‐seedlings over 19 months of growth.

All statistical analyses were done within the R*‐*framework v. 4.5.2. (R Core Team 2025). We used linear mixed models with gaussian errors and generalized linear mixed models (package glmmTMB, Brooks *et al*. 2017) for models with other error types (Table 1). Model assumptions were checked with the DHARMa‐package (Hartig 2024). The population of the mother plants and mother plant identity (n = 111) were included as random factors. *P*‐values were obtained using the anova and ANOVA (package car, Fox & Weisberg 2019) commands. Marginal means were calculated using the emmeans‐package (Lenth & Piaskowski 2025).

**Table 1:** Results of linear mixed models (LMM, F‐values) or generalized linear mixed models (GLMM, χ^2^‐values) of the effect of crossing treatments on reproduction of the F_0_‐plants, proportion of seeds germinating and F_1_‐seedlings surviving.

trait	mother taxon (MT)		pollination treatment (PT)		MT × PT		Model, error type
	F or χ^2^	*P*	F or χ^2^	*P*	F or χ^2^	*P*	
Fruits per flower	3.35	0.067	0.80	0.700	0.59	0.746	GLMM, binomial
Number of seeds per fruit	4.80	0.028	0.34	0.842	0.44	0.802	GLMM, negative binomial
Seed mass	0.05	0.829	6.02	0.004	0.80	0.454	LMM, gaussian
Seed yield per flower							
Zero‐inflated model	5.61	0.018	4.72	0.094	0.88	0.646	GLMM hurdle, zero‐inflated negative binomial
Conditional model	2.49	0.114	10.32	0.006	0.87	0.646	
Proportion seeds germinating	2.76	0.010	2.40	0.301	0.03	0.985	GLMM, betabinomial
Survival of F_1_‐plants	5.36	0.021	18.00	<0.001	7.74	0.021	GLMM, binomial

Seed yield per flower = Fruits per flower × seeds per fruit × mass per seed. The population of the mother plants and mother plant identity (n = 111) were included as random factors.

As an estimate of the cumulative effect of the cross treatments on reproduction, we calculated seed yield per flower as the product of the fruit/flower ratio × seed number per fruit × seed mass. Due to the high number of zeros in the data, we used a zero‐inflated negative‐binomial hurdle model. The hurdle model consisted of two parts: The zero‐inflated model described the effect of the treatments on the probability that no seeds were produced by a flower, while the conditional model described the effect on the seed yield per fruit. Standard errors were obtained by the error propagation formula for the standard error of a product of two means (Buonaccorsi & Liebhold 1988).

For the analysis of the second part of the experiment, we used linear and generalized linear mixed models which included the identity of the F_1_‐plants used as mother plants (n = 69) as a random factor to analyse the effects of the different pollination treatments on the following traits (Table 2): Proportion of flowers of the F_1_‐plants developing into a fruit, the number of seeds per fruit, the mean mass of the individual seeds, the proportion of seeds that germinated and the survival of the F_2_‐seedlings over 5 months of growth. Seed yield per flower was calculated in the same way as for the previous generation.

**Table 2:** Results of linear mixed models (LMM, F‐values) or generalized linear mixed models (GLMM, χ^2^‐values) of the effect of crossing treatments on reproduction of the F_1_‐parents, proportion of seeds germinating and the survival of F_2_‐seedlings.

trait	pollination treatment		model, error type
	F or χ^2^	*P*	
Fruits per F_1_‐flower	27.06	<0.001	GLMM, betabinomial
Number of seeds per fruit	41.22	<0.001	GLMM, negative binomial
Seed mass	0.57	0.903	LMM, gaussian
Seed yield per flower			
Zero‐inflated model	62.47	<0.001	GLMM hurdle model, zero‐inflated negative binomial
Conditional model	12.12	0.007	
Proportion of seeds germinating	5.25	0.154	GLMM, betabinomial
Survival of F_2_‐seedlings	8.41	0.038	GLMM, betabinomial

The identity of the F_1_‐individual used as a mother plant (n = 69) was included as a random factor in the model.

To estimate the effect of the different pollination treatments over the course of the whole experiment, we calculated a cumulative fitness measure as the product of the following fitness components: Fruits per flower of the F_0_ × seeds per fruit × proportion of seeds germinating × survival of F_1_‐plants × fruits per flower of the F_1_ × seeds per fruit × proportion of seeds germinating × survival of F_2_‐plants.

## RESULTS

Of the 144 crosses carried out with the F_0_‐parent plants, 106 produced fully developed seeds. There was only weak evidence that the proportion of flowers that developed into fruits was higher for the *S. rosacea* ssp. *rosacea* (82.2%) than for the subspecies *sponhemica* (67.8%; Table 1), but the number of seeds developed per fruit was much higher for *S. rosacea* (108.4) than for *S. sponhemica* (66.4). However, there was little evidence for an effect of crossing type on the proportion of flowers developing into a fruit or the number of seeds per fruit (Fig. 2a,b). The mass of the seeds was similar for WPC and BPC, but lower for BTC (Fig. 2c). Seed mass was similar for *S. rosacea* (28.6 μg) and *S. sponhemica* (28.0 μg). Seed yield per F_0_‐flower as a measure of overall reproductive success was higher for BPC than WPC and BTC crosses (Fig. 2d), indicating heterosis, and was 70% higher for *S. rosacea* than for *S. sponhemica*. Germination of the seeds was also higher for *S. rosacea* (69.9%) than for *S. sponhemica* (57.4%), but there was little evidence for an effect of crossing type (Fig. 2e; Table S1). For none of the reproductive traits was there clear evidence for an interaction between the effects of mother species and crossing type (Table S1).

**Fig. 2:**
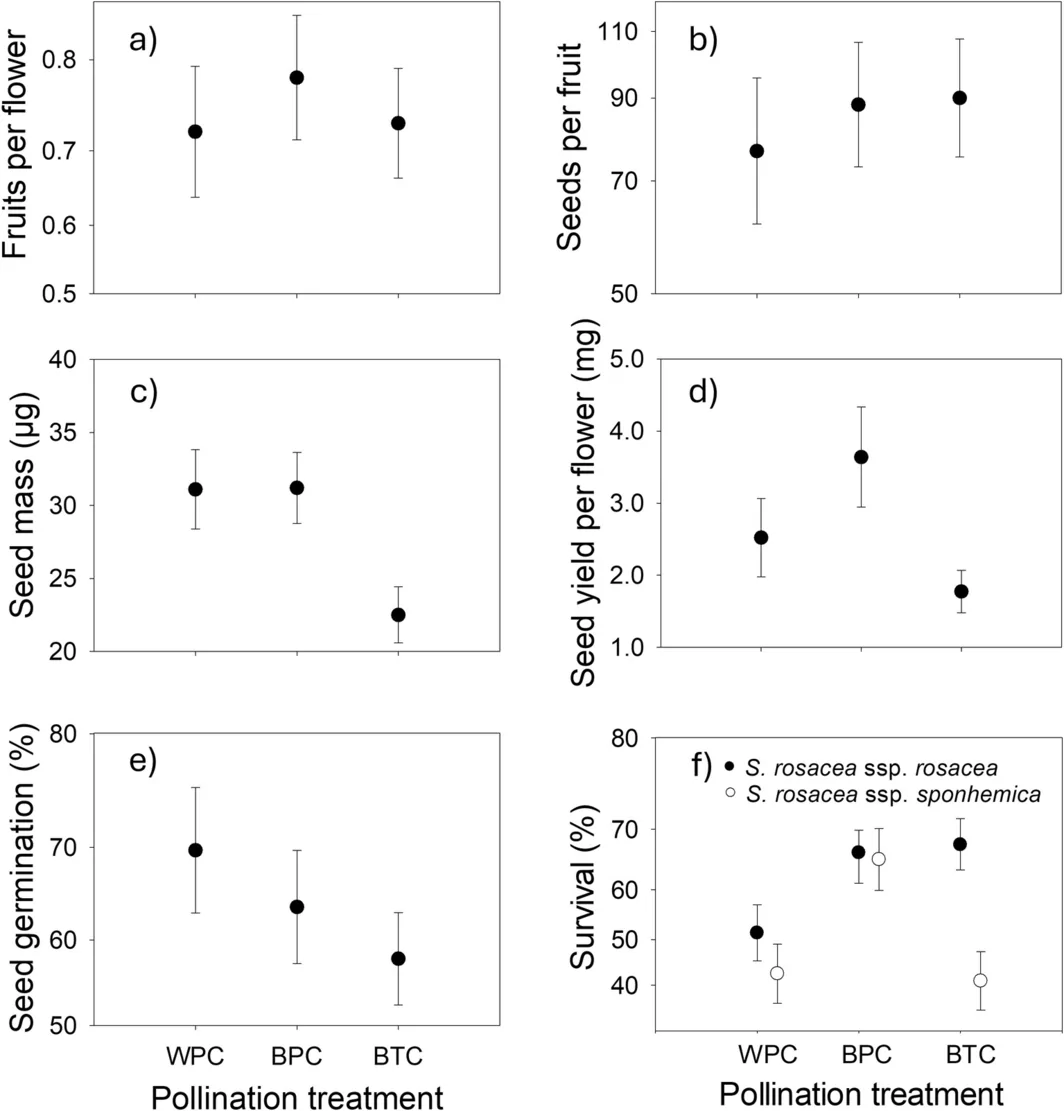
The effect of crossing treatments applied to *S. rosacea* and *S. sponhemica* on various traits. (a) Proportion of flowers of the F_0_ that produced a fruit, (b) number of seeds per fruit, (c) seed mass, (d) seed yield per flower, (e) seed germination, (f) survival of F_1_‐seedlings over 19 months. WPC—within‐population crosses; BPC—between‐population crosses; BTC—between‐taxon crosses.

By contrast, seedling survival depended on the combination of mother species and crossing type (Fig. 2f). Survival of seedlings from BPC was higher than that of WPC (+33%), indicating heterosis, and this difference was similar for both taxa. By contrast, after BTC, survival was much higher for offspring of a *S. rosacea* mother than for that of a *S. sponhemica* mother.

The different types of crosses strongly influenced both the fruit/flower ratio of the offspring of both taxa and the number of seeds per fruit produced (F_1_‐plants; Table 2 and Fig. 3a,b). The proportion of flowers developing into a fruit (−52%) and the number of seeds per fruit (−63%) were much lower for offspring of between‐population crosses (BPC → WPC) than for offspring of within‐population crosses (WPC → WPC). Reproduction of offspring of between‐taxon crosses (BTC → WPC) was extremely low, with outbreeding depression of 84% for the fruit/flower ratio and 73% for the number of seeds per fruit (s. Fig. 3a,b). However, seed mass was similar across treatments (Fig. 3c). Seed yield per flower as a measure of overall reproductive success was extremely low for offspring of between‐taxon crosses in comparison to that of within‐population crosses (−95%, Fig. 3d), indicating hybrid sterility and reproductive isolation. Values for backcrosses were similarly low. By contrast, seed germination was not clearly influenced by pollination treatment (Table 2; Fig. 3e). However, survival of the seedlings was higher for F_2_‐offspring of BPC (BPC → WPC) than for offspring of WPC (WPC → WPC), indicating heterosis (Fig. 3f). Combined over the two generations studied, the relative cumulative fitness of the different crosses was WPC: 1, BPC: 0.37; BTC: 0.05.

**Fig. 3:**
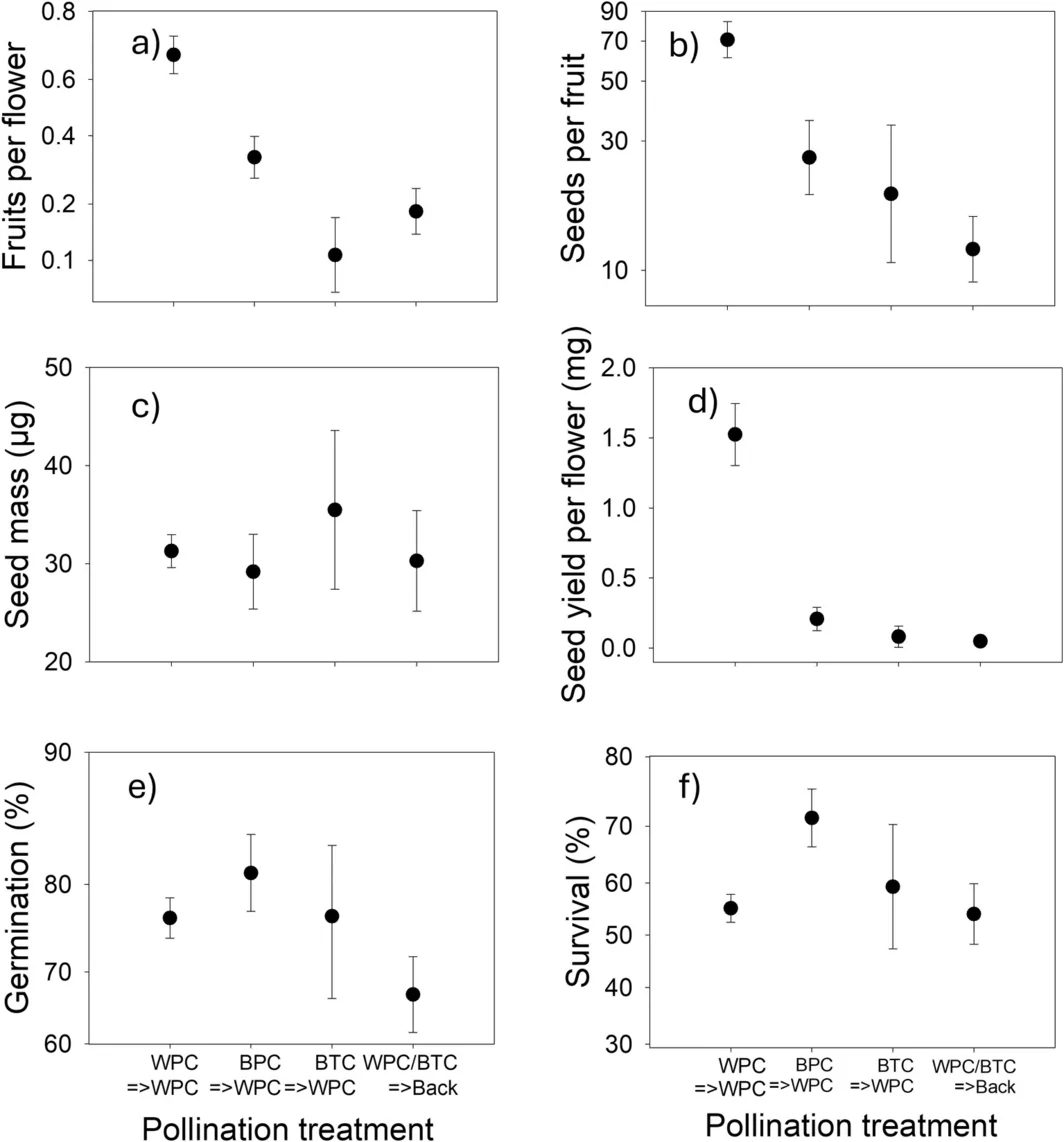
The effect of various pollination treatments on traits of the F_1_‐plants used as parents and the F_2_‐generation of *S. rosacea* and *S. sponhemica*. (a) Proportion of flowers of the F_1_‐plants producing a fruit, (b) number of seeds per fruit, (c) seed mass, (d) seed yield per flower, (e) seed germination, (f) survival of F_2_‐seedlings. WPC → WPC: Crosses between F_1_‐plants that had resulted from crosses within the same parent population; BPC → WPC: Crosses between F_1_‐plants that had been produced by crosses between the same two populations; BTC → WPC: Crosses between F_1_‐plants that had been produced by between‐cytotype crosses; Back: backcrosses between F_1_‐plants that had been produced by within‐population crosses with plants that had been produced by between‐cytotype crosses, and one of whose parents was from the same population as the WPC‐individual.

## DISCUSSION

In our crossing experiment, effects of the treatments varied strongly among different components of fitness. We found in crosses between populations of the same taxon heterosis for some traits and outbreeding depression for others. Seed yield of BPC and survival of offspring were higher than those of WPC, indicating realized genetic load (Bertorelle *et al*. 2022) in the isolated rock populations of both taxa. However, BPC offspring showed outbreeding depression for reproduction. BTC offspring was nearly completely infertile, indicating strong reproductive isolation between the two taxa.

### Effects of crosses between populations of the same taxon on fitness components

The net effect of crosses between populations on components of fitness depends on the balance between heterosis and outbreeding depression (Hufford & Mazer 2003; Oakley *et al*. 2015). In both *Saxifraga‐*taxa, we found heterosis in early traits like seed yield of the F_0_ and survival of the F_1_‐seedlings, while there was strong outbreeding depression in reproduction of the F_1_. Seed yield per flower of between‐population crosses was 44% higher than that of within‐population crosses and survival 33% higher, indicating considerable realized genetic load in the studied populations of both taxa. The populations of the *Saxifraga* taxa are strongly fragmented, and their populations have been isolated for a long time (Decanter *et al*. 2020). These conditions favour genetic drift that results in a loss of genetic diversity, increased homozygosity and the expression of recessive alleles with negative effects as well as genetic differentiation among populations (Fischer & Matthies 1998; Kéry *et al*. 2000; Waller 2015; Perrier *et al*. 2020). Crosses between populations of the same taxon resulted in heterosis and alleviated the detrimental effects of genetic load accumulated in the populations. This increase in performance is attributed to the masking of deleterious recessive alleles after mixing plants from different populations that had lost genetic variation through drift (Tallmon *et al*. 2004). Similar heterosis in early traits due to interpopulation crosses has been found in other rare species (Oostermeijer *et al*. 1995; Sheridan & Karowe 2000; Pickup *et al*. 2013).

The genetic diversity of populations of one of the two *Saxifraga*‐taxa (*S. sponhemica*) has been studied using RAPD‐markers (Walisch *et al*. 2015). Mean molecular genetic diversity (He) within the populations was 0.27, and this value did vary little among populations, suggesting only a moderate amount of loss of genetic diversity in the isolated populations (Walisch *et al*. 2015). However, this does not necessarily contradict the conclusion that there has been fixation of recessive deleterious alleles due to drift because there is not always a strong correlation between‐population fitness and molecular genetic diversity (Leimu *et al*. 2006; Teixeira & Huber 2021). Moreover, within individual genetic variation, heterozygosity may have decreased more strongly in the isolated populations by drift than the genetic variation among individuals (Siol *et al*. 2008; Volis & Zhang 2010), possibly facilitated by geitonogamy within the large individuals and the close relatedness of neighbouring individuals (Walisch *et al*. 2015).

Crosses between the parental populations resulted in both taxa in strongly reduced fruit set and seed production of the F_1_‐generation compared to within‐population crosses, indicating strong outbreeding depression. Seed yield per flower as a measure of overall reproductive success was reduced by 95%. The reduced fitness of intraspecific hybrids can be attributed to the breakup of coadapted gene complexes or chromosomal rearrangements (Hufford *et al*. 2012; Oakley *et al*. 2015). The negative effects of the breakup of coadapted gene complexes through interpopulation crosses may be masked in the F_1_ by heterosis and only become apparent through segregation in the F_2_ and following generations (Lynch 1991; Hufford & Mazer 2003; Whitlock *et al*. 2013; Frankham 2016), although there are other examples of outbreeding depression in the F_1_ (Fischer & Matthies 1997; Hufford *et al*. 2012; Oakley *et al*. 2015). The observed pattern of heterosis in early traits and strong outbreeding depression in reproduction of the F_1_ in the present experiment fits this predicted pattern if the reduced fertility of the interpopulation hybrids in the *Saxifraga* was due to a failure of embryos to develop. However, at later stages, there was no evidence for outbreeding depression in germination of the F_2_, and there was even heterosis in early survival. Overall, our results therefore support the notion that the effects of interpopulation crosses (both heterosis and outbreeding depression) are usually stage specific (Fischer & Matthies 1997; Sheridan & Karowe 2000; Becker *et al*. 2006; Walisch *et al*. 2012; Bürli *et al*. 2024).

Outbreeding depression after interpopulation crosses is not universal (Walisch *et al*. 2012; Pickup *et al*. 2013; Shi *et al*. 2018; Bürli *et al*. 2024; and the many examples of high‐yield hybrid crops). It will depend on population history, population size and mating system, and tends to increase with the geographical, ecological and genetic distance between populations (Galloway & Etterson 2005; Grindeland 2008; Forrest *et al*. 2011; Hufford *et al*. 2012). A meta‐analysis of the effects of outcrossing on inbred populations that were considered to have a low risk of outbreeding depression concluded that the effects on reproductive fitness in the F_1_ and later stages were mostly positive (Frankham 2016). In the present case, the strong outbreeding depression indicates substantial genetic differentiation among the *Saxifraga populations* due to a long history of genetic drift.

### Effects of crosses between the two taxa

Seed yield per flower after between‐taxon crosses (BTC) was 30% lower than that of within‐population crosses, indicating early outbreeding depression after crosses between the different taxa. The reduction in reproduction due to BTC was less pronounced than observed in studies that have reported reproductive failure (hybrid seed inviability; Burton & Husband 2001; Köhler *et al*. 2010; Morgan *et al*. 2021; Lattier & Contreras 2022). The frequent failure to produce viable seeds has been attributed to the malfunction of the endosperm after interploidy crosses, called triploid block after the simplest case of interploidy crosses, that is 2× by 4× crosses producing triploids. The normal 2:1 ratio of maternal (m) to paternal (p) contribution to the endosperm is thought to be critical for seed maturation in many species, and deviations from this ratio lead to reproductive failure (Köhler *et al*. 2010; Sonnleitner *et al*. 2013). However, recently increasing evidence has accumulated that interploidy crosses may produce viable seeds and that interploidy introgression in ploidy‐variable species is a frequent phenomenon (Peckert & Chrtek 2006; Bartolić *et al*. 2024; Yakushiji *et al*. 2024). Interploidy crosses may be particularly successful between higher level polyploids because they may result in smaller deviations from the optimal 2 m:1p ratio in the endosperm than in triploids (Koutecký *et al*. 2011; Sonnleitner *et al*. 2013). Our observation of successful seed set after 6× by 8× crosses in *Saxifraga* supports the hypothesis that reproductive barriers between different cytotypes may be more relaxed than previously thought.

The direction of the interploidy crosses strongly influenced the survival of the F_1_‐plants. Maternal excess crosses (8× *S. rosacea* mothers) survived much better than paternal excess crosses. Differences in the fitness of the F_1_ depending on the direction of the cross have been found in other studies, but with no clear advantage for either maternal or paternal excess crosses (Burton & Husband 2001; Morgan *et al*. 2021 and citations therein; Lattier & Contreras 2022). Between‐taxon crosses resulted in much higher survival of F_1_‐plants from *S. rosacea* mothers than from *S. sponhemica* mothers. Moreover, we found a much higher seed yield per flower of the F_0_‐generation for *S. rosacea* plants and a higher germination of seeds produced by *S. rosacea* mothers both by interploidy and intraploidy crosses, indicating a generally higher number and quality of the seeds produced by *S. rosacea* mothers.

The seed yield of both the BTC F_1_‐plants and of their backcrosses with the parent taxa was extremely low, indicating strong outbreeding depression (95% and 97%), but they were not completely sterile. The crosses between 6× *S. sponhemica* and 8× *S. rosacea* presumably resulted in heptaploids. Cytotypes with odd ploidy levels are typically infertile or show strongly reduced fertility due to meiotic irregularities and a failure to produce gametes (Bruneau and Anderson 1988; Ramsey & Schemske 1998; Burton & Husband 2001; Ramsey *et al*. 2011), but there are exceptions (Gajdošová *et al*. 2023; Yakushiji *et al*. 2024). The extremely low fertility of the F_1_‐plants from heteroploidy crosses indicates that the two taxa are reproductively isolated. It is thus very unlikely that there would be introgression or that hybrid populations are formed. This is further confirmed by the fact that no mixed populations of both *Saxifraga* taxa have ever been found (Decanter *et al*. 2020).

## CONCLUSIONS

Polyploids may experience a slower loss of heterozygosity due to habitat fragmentation and isolation than diploids and thus suffer less from genetic erosion (Moody *et al*. 1993; Brown & Young 2000; but see Aguilar *et al*. 2008), and they may therefore face a lower risk of local extinction (Plue *et al*. 2018). Moreover, recessive deleterious mutations may be more strongly masked in polyploids (Lande & Schemske 1985; Grindeland 2008), reducing the risk of inbreeding depression, but the empirical evidence is inconclusive (Clo & Kolář 2022). We found considerable realized genetic load in the isolated populations of both studied polyploid taxa, supporting the notion that polyploids may face similar risks due to genetic drift as diploids (Pickup & Young 2008).

Both studied *Saxifraga* taxa are rare in Central Europe and occur only on rocks or drystone walls. *S. rosacea* ssp. *sponhemica* is even an extremely rare central European endemic (Walisch *et al*. 2015) threated by anthropogenic habitat destruction and natural succession leading to tree canopy closure (Decanter 2021). To reduce the risk of regional extinction, the establishment of new populations at suitable sites should be considered as a conservation measure, in particular, for *S. rosacea* ssp. *sponhemica*. Opportunities for creating new populations exist at rocks and drystone walls. The heterosis observed after between‐population crosses indicates that mixing rosettes or seeds from different source populations could increase the chances of success for the establishment of new populations of both taxa. However, over two generations, we found an outbreeding depression of c. 63% in comparison to crosses within populations due to strongly reduced reproduction of the F_1_. Our results thus suggest that, in the context of restoration projects, caution is necessary when increasing gene flow between long‐term isolated populations or when maximizing genetic variation in newly created populations by using mixtures of seeds from different populations.

However, the observed outbreeding depression might be acceptable when creating new populations as it has to be balanced against the possible positive effects of higher genetic diversity and more opportunities for the selection of better adapted genotypes (Newman & Tallmon 2001; Bell *et al*. 2019; Brisset *et al*. 2025). This could be particularly important in the face of an increased risk of drought in rock habitats due to climate change. The negative effects of outbreeding observed in the F_1_ were partly compensated by higher survival of the F_2_‐plants, and the negative effects of outbreeding may decrease in further generations (Richards 2000; Frankham 2015; Onorato *et al*. 2024).

Recently, evidence has accumulated that crosses between cytotypes with different ploidy levels are more frequent than previously thought and hybrids are not always sterile (Bartolić *et al*. 2024). However, the nearly complete infertility of the F_1_‐plants resulting from crosses between the octoploid S. *rosacea* ssp. *rosacea* and the hexaploid S. *rosacea* ssp. *sponhemica* strongly supports the advice that management measures that might result in heteroploid crosses should be avoided (Severns & Liston 2008; Frankham *et al*. 2011; Kramer *et al*. 2018). Moreover, the sterility of the hybrids suggest that the two taxa should be treated as separate species. The failure of hybrids between two taxa to produce offspring is a classical argument of the biological species concept for delimiting species (Briggs & Walters 1997). The classification as two species is further supported by differences between the two taxa in their morphology (Webb 1963), in their ecological niches and in their patterns of distribution with no contact zones (Decanter *et al*. 2020). We thus suggest that the accepted names for the two taxa should be *Saxifraga rosacea* Moench and *Saxifraga sponhemica* C.C.Gmel.

## AUTHOR CONTRIBUTIONS

GC and DM designed the study, LD performed the experiments, GC, DM and LD analysed the data and wrote the manuscript.

## Supporting information


**Table S1.** Location of the 30 populations of *Saxifraga rosacea* and *Saxifraga sponhemica* used for the crossing experiments.

**Data S1.** Matrix showing which populations were crossed to produce between‐population crosses for *Saxifraga rosacea* ssp. *rosacea* (BPC SRR) and *S. rosacea* ssp. sponhemica (BPC SRS), as well as crosses between the two taxa (BTC).

## Data Availability

The data that support the findings of this study are openly available in the Zenodo repository: https://doi.org/10.5281/zenodo.23010934
