# Supplementary material for: Effects of crossing distance on components of fitness in two rare rock plants
Source: Plant Biol (Stuttg). 2026 Oct 7;28(7):2285–94. doi: 10.1111/plb.70297 (PMC13750316; doi:10.1111/plb.70297)
Supplement: Supplementary file 1 [file PLB-28-2285-s001.docx]

**Supporting information 1**

**Table S1.** Location of the 30 populations of *Saxifraga rosacea* and *Saxifraga sponhemica* used for the crossing experiments*.*

| Subspecies | Region | Population | Lat. | Long. |  |
| --- | --- | --- | --- | --- | --- |
| *S. rosacea* | Jura | Baume-les-messieurs | 46.69 | 5.64 |  |
|  | Vosges | Hartmannswillerkopf | 47.86 | 7.17 |  |
|  | Central | Bronn | 49.73 | 11.46 |  |
|  | Germany | Gräfenwarth | 50.53 | 11.71 |  |
|  |  | Harzer-Hexen-Stieg | 51.76 | 10.84 |  |
|  |  | Lochbauer | 50.53 | 12.17 |  |
|  |  | Neuhaus | 49.63 | 11.55 |  |
|  |  | Neutal | 49.06 | 11.57 |  |
|  |  | Pegnitz | 49.69 | 11.55 |  |
|  |  | Pfaffenhofen | 49.64 | 11.53 |  |
|  |  | Pöritzsch | 50.50 | 11.73 |  |
|  |  | Röttis | 50.54 | 12.17 |  |
|  |  | Seeweiher | 49.69 | 11.54 |  |
|  |  | Velden | 49.61 | 11.52 |  |
|  |  | Wied | 49.51 | 11.50 |  |
| *S. sponhemica* | Jura | Arbois | 46.88 | 5.81 |  |
|  |  | Salin | 46.93 | 5.92 |  |
|  | Ardennes | Bouillon | 49.80 | 5.07 |  |
|  | Eifel | Gerolstein | 50.20 | 6.63 |  |
|  |  | Hammerstein | 49.69 | 7.30 |  |
|  | Nahetal | Frauenberg | 49.68 | 7.29 |  |
|  | Czech | Blešno | 50.48 | 13.90 |  |
|  | Republic | Borec | 50.52 | 13.99 |  |
|  |  | Milešov | 50.53 | 13.95 |  |
|  |  | Tétinské | 49.57 | 14.06 |  |
|  |  | Voškov | 49.92 | 14.18 |  |
|  | Oesling | Bettel | 49.92 | 6.22 |  |
|  |  | Kautenbach | 49.95 | 6.02 |  |
|  |  | Michelau | 49.89 | 6.12 |  |
|  |  | Unterschlinder | 49.92 | 6.07 |  |
|  |  |  |  |  |  |
